# Discrimination of Breast Cancer with Microcalcifications on Mammography by Deep Learning

**DOI:** 10.1038/srep27327

**Published:** 2016-06-07

**Authors:** Jinhua Wang, Xi Yang, Hongmin Cai, Wanchang Tan, Cangzheng Jin, Li Li

**Affiliations:** 1 Department of Radiology, Affiliated Nanhai Hospital of Southern Medical University, Foshan 528200, Guangdong, China; 2 School of Computer Science and Engineering, South China University of Technology, Guangzhou 510006, Guangdong, China; 3 Sun Yat-sen University Cancer Center; State Key Laboratory of Oncology in South China; Collaborative Innovation Center for Cancer Medicine, Guangzhou 510060, Guangdong, China

## Abstract

Microcalcification is an effective indicator of early breast cancer. To improve the diagnostic accuracy of microcalcifications, this study evaluates the performance of deep learning-based models on large datasets for its discrimination. A semi-automated segmentation method was used to characterize all microcalcifications. A discrimination classifier model was constructed to assess the accuracies of microcalcifications and breast masses, either in isolation or combination, for classifying breast lesions. Performances were compared to benchmark models. Our deep learning model achieved a discriminative accuracy of 87.3% if microcalcifications were characterized alone, compared to 85.8% with a support vector machine. The accuracies were 61.3% for both methods with masses alone and improved to 89.7% and 85.8% after the combined analysis with microcalcifications. Image segmentation with our deep learning model yielded 15, 26 and 41 features for the three scenarios, respectively. Overall, deep learning based on large datasets was superior to standard methods for the discrimination of microcalcifications. Accuracy was increased by adopting a combinatorial approach to detect microcalcifications and masses simultaneously. This may have clinical value for early detection and treatment of breast cancer.

Breast cancer is the most common cancer in women worldwide and the second leading cause of female cancer deaths. Mammography is the most efficient method for screening breast cancer and can reduce breast cancer mortality^1^,^2^,^3^. One of the main early symptoms on mammograms is the appearance of microcalcifications, whose diameter range from 0.1 to 1 mm^2^,^4^,^5^,^6^. Early detection and accurate identification of malignant microcalcifications can facilitate early detection, diagnosis and timely treatment of breast cancer^2^,^5^,^7^. However, due to the small size and low contrast compared to the background of images, it is difficult and time for radiologists to make objective and accurate evaluation of microcalcifications^8^,^9^,^10^,^11^. The problem is especially challenging for inexperienced radiologists when facing enormous numbers of mammograms generated in widespread screening^9^,^10^,^11^. Consequently, there is a need to develop helpful automated tools to overcome these problems and improve diagnostic performance of breast cancer. Advances in computer technologies allow comprehensive and objective analysis of diagnostic features in microcalcifications and masses^12^,^13^,^14^. Meanwhile, by efficiently analyzing large numbers of images, computer-based methods can minimize intra- and inter-observer performance variability^12^,^13^,^14^,^15^. Through the automatic identification and classification of microcalcifications, computer-based methods can be proposed to aid early detection and diagnosis^13^,^15^.

A wide variety of machine learning classifiers have been developed for early diagnosis of breast cancer^16^,^17^,^18^. The widely used techniques are based on support vector machines (SVM)^18^,^19^,^20^, k-nearest neighbor (KNN) method^21^,^22^ and linear discriminant analysis (LDA)^23^,^24^. However, the discriminative power of these methods is limited due to the computational costs of identifying definitive features for subset characterization and optimization. Deep learning is a relatively new method in the field of artificial intelligence and machine learning technologies^25^,^26^,^27^,^28^,^29^,^30^,^31^. This approach has achieved considerable successes in multiple applications, including medical research. Deep convolutional neural networks were employed to medical image classification^31^; deep belief nets and active learning were presented for multi-level gene and miRNA feature selection^25^; convolutional neural networks were used to demonstrate an explicit gradient for feature complexity in the ventral pathway of the human brain^26^; deep learning was applied to determine the sequence specificities of DNA and RNA-binding proteins for identifying causal disease variants^27^; superpixel and deep learning were used for automatic vaginal bacteria segmentation and classification^28^; some deep learning-based latent feature representations are proposed for diagnosis of Alzheimer’s disease and its prodromal stage, mild cognitive impairment (MCI), such as stacked auto-encoder and deep boltzmann machine^32^,^33^. However, only few works have explored deep learning methods to address the automatic classification of identified lesions on mammography. A nice learning framework for breast cancer diagnosis in mammography by convolutional neural networks was reported^34^. The tested data were preprocessed images. A convolutional sparse autoencoder was proposed for mammographic texture scoring^35^.

Deep learning comprises a neural network with multiple hidden layers that enhances the recognition accuracy of images, audio and other data types; thereby increasing its versatility for capturing representative features. Deep learning outperforms other state-of-the-art methods in many areas and has solved complicated pattern recognition problems, especially in big data situations^36^,^37^,^38^,^39^. Stacked denoising autoencoder model is one of the most successful deep learning strategies. The deep architecture can be used to discover latent or hidden representation efficiently inherent in the low-level features from modalities, and ultimately to enhance classification accuracy. In this study, with a stacked denoising auto-encoder, an innovative deep learning-based model was employed to retrospectively analyze a large sample of microcalcifications with or without masses on mammography. Its performance and accuracy in classifying and discriminating breast lesions were compared with benchmark models.

## Results

The training group consisted of 1000 images, including 677 benign and 323 malignant lesions. The test group consisted of 204 images, including 97 benign and 107 malignant lesions. Table 1 shows the histopathological distributions of the lesions in both groups. Data about microcalcifications and suspicious breast masses were extracted through image segmentation. Both statistical and textural features were used to classify image features and obtain comprehensive characterization of the microcalcifications and masses. A total of 41 quantitative measurements were recorded for each patient. Detailed information is provided in the Appendix File S1. Fifteen microcalcifications features and twenty-six breast masses features were feed into the comparative classifiers, including SVM, LDA, and KNN. These features were selected since they have been shown to improve the performance of standard machine learning classifiers in earlier researches on breast lesions^18^,^19^,^23^,^24^,^34^,^40^,^41^.

**Figure 1:**
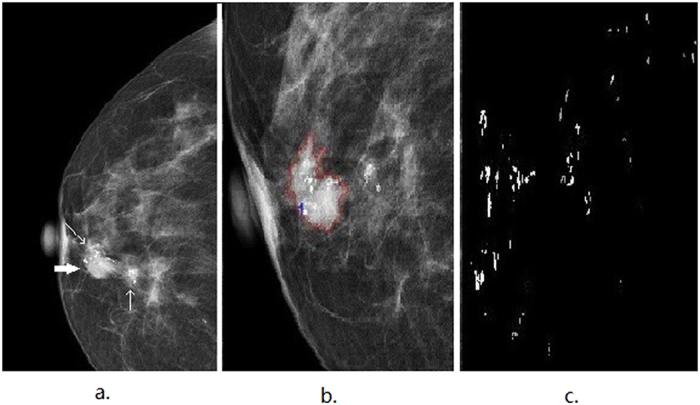
An illustrative example showing segmentation of microcalcifications and breast mass in a mammogram of the left breast of a 60-year-old patient with invasive ductal carcinoma. (**a**) The craniocaudal (CC) view shows focal clustered microcalcifications (indicated by thin arrows) and an irregular circumscribed mass (indicated by a thick arrow). (**b**) The suspicious mass is automatically delineated within the red curve. (**c**) the segmented microcalcifications detected in (**b**) are used to characterize the features.

**Figure 2:**
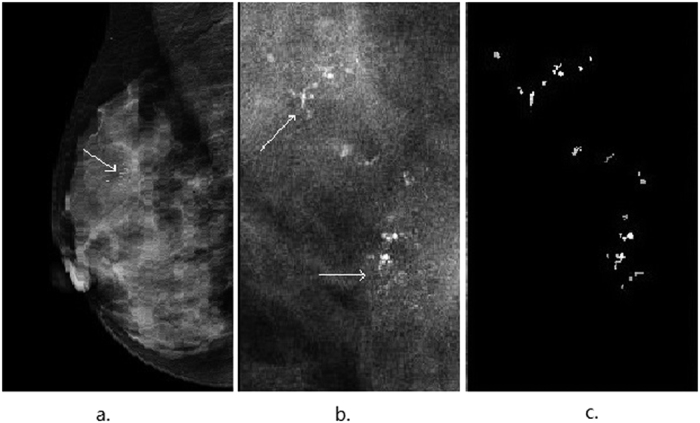
An illustrative example showing segmentation of microcalcifications in a mammogram of the left breast of a 56-year-old patient with ductal carcinoma *in situ*. (**a**) The mediolateral oblique (MLO) view shows clustered coarse and low density microcalcifications (indicated by thin arrows). (**b**) The image shows the region of suspicious microcalcifications(indicated by thin arrows). (**c**) The segmented microcalcifications from (**b**) are used to characterize the features.

**Figure 3:**
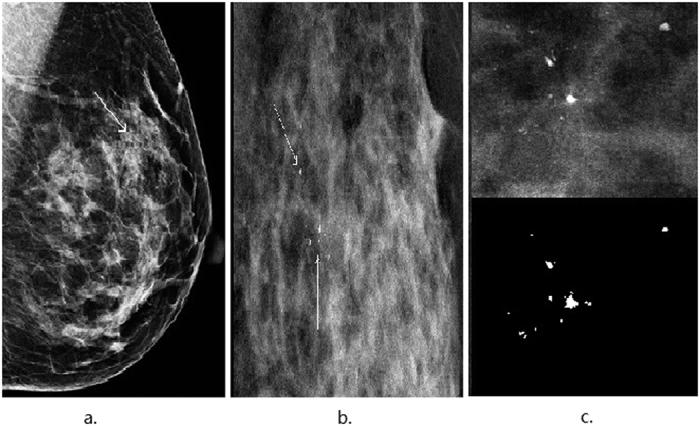
An illustrative example showing segmentation of microcalcifications in a mammogram of the right breast of a 49-year-old patient with fibrocystic changes. (**a**) The focal microcalcifications (indicated by thin arrows) appear low contrast compared with the dense background in the mediolateral oblique (MLO) view. (**b**) The region of suspicious microcalcifications is indicated by thin arrows. (**c**) A zoomed-in view of (**b**) highlights the segmented microcalcifications.

Figure 1 illustrates an automatic detection and segmentation pipeline to identify suspicious microcalcifications and masses in the left breast of a 60-year-old patient with invasive ductal carcinoma. The microcalcifications were extracted from the raw data to delineate the image characteristics (Fig. 1(b)). Figure 2 shows that this method could accurately detect and extract suspicious microcalcifications from the background of a low-density image showing the left breast of a 56-year-old patient with ductal carcinoma *in situ*. This demonstrated the high accuracy and robustness of the image segmentation pipeline. Figure 3 shows the image of the right breast of a 49-year-old patient with fibrocystic changes in which the focal microcalcifications appear low contrast compared with the high-density background. Extraction of suspicious microcalcifications is a challenging task, however, these results demonstrated that our segmentation model was able to accurately identify and extract microcalcifications from the images to facilitate characterization.

In order to evaluate the performance and discriminative power of the deep learning model (DL), quantitative measurements for overall classification accuracy (acc), sensitivity, specificity and the area under the receiver operating characteristic (ROC) curve (AUC) were calculated as follows:


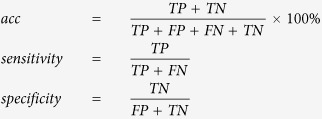


where *TP*, *FN*, *TN* and *FP* represent the true positives, false negatives, true negatives and false positives, respectively.

**Figure 4:**
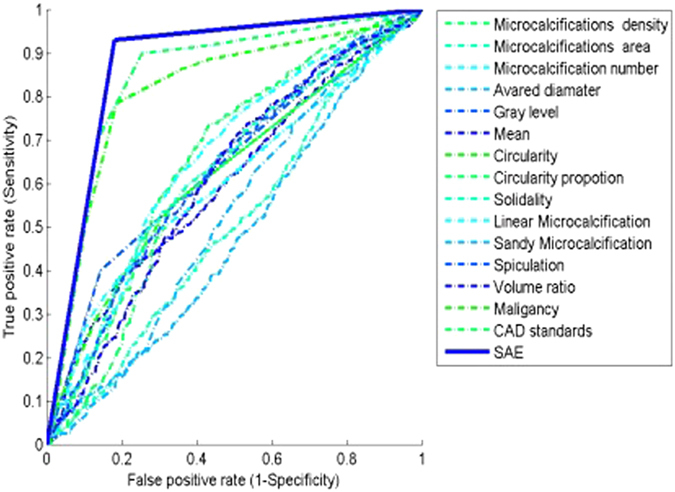
ROC curves for selected microcalcification features. The ROC curves compare the discriminative performances of individual features versus combinations of features.

Previous reports have suggested that the discriminative performances of classifiers can be increased through comprehensive characterization of microcalcifications as opposed to characterization of individual features. In agreement with these reports, our deep learning-based model achieved similar outcomes, as demonstrated by the ROC curves in Fig. 4. Therefore, this approach was used in the following experiments.

Three scenarios for discriminating between malignant and benign lesions were examined: microcalcifications alone; breast masses alone; and microcalcifications and breast masses in combination. The primary aims of the three scenarios were to investigate the discrimination power of microcalcifications, masses or their combination in differentiation of the lesions types. The results were compared to those of SVM, KNN and LDA benchmark classifiers.

The structure of a SAE network is decided by the size of the input layer, the number of hidden layers, and the number of hidden units in each hidden layer. Through the experiments, the data of microcalcifications alone was used as the input in the first scenario; the data of breast masses alone was served as the input in the second scenario; and the data of microcalcifications and breast masses in combination was served as the input in the third scenario. We used the SAE model to classify malignant and benign lesions in three scenarios. The optimal hyper parameters for the three scenarios were estimated by 10 fold cross-validation on training group. For the first scenario, the trained architecture consisted of two hidden layers, and the number of hidden units in each hidden layer was [200, 200], respectively. For the second scenario, the trained architecture consisted of two hidden layers, and the number of hidden units in each hidden layer was [200, 200], respectively. For the third scenario, the trained architecture consisted of two hidden layers, and the number of hidden units in each hidden layer was [400, 400].

**Figure 5:**
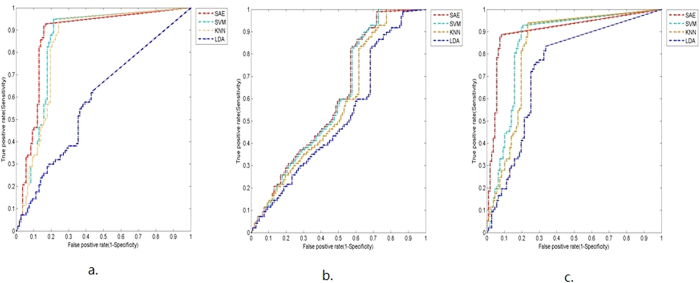
ROC curves comparing the discriminative performances of the four classification models. The three scenarios show the ROC curves based on the following classifications: (**a**) Microcalcifications alone (15 segmentation features). (**b**) Breast masses alone (26 segmentation features). (**c**) Microcalcifications plus breast masses (41 segmentation features).

In the first scenario, image segmentation yielded 15 features. The overall accuracies were 85.8%, 83.8%, 58.8% and 87.3% for the SVM, KNN, LDA and DL models, respectively. The DL model also achieved the highest specificity and AUC values (0.82 and 0.87, respectively). The results are summarized in Table 2; the ROC curves in Fig. 5(a) provided visual comparisons between the models.

In the second scenario, based on breast masses alone, image segmentation yielded 26 features. The results are summarized in Table 3, and the ROC curves are shown in Fig. 5(b). The overall accuracies were markedly lower in all of the models, at 61.3%, 58.8%, 53.4% and 61.3% for SVM, KNN, LDA and DL respectively. Furthermore, the performance of the DL model was only marginally higher than that of the SVM model. Despite this finding, the sensitivity of the model was approximately 100%, indicating that patients who tested positive all had breast masses. As such, this method may facilitate diagnosis in benign cases; however, it may not serve as a valid diagnostic tool in clinical practice.

In the third scenario, based on a combinatorial approach by analyzing microcalcifications and breast masses simultaneously, image segmentation yielded 41 features. The overall accuracies were 85.8%, 84.3%, 74.0% and 89.7% for the SVM, KNN, LDA and DL models, respectively. Furthermore, the DL model achieved the highest specificity and AUC values (0.90 and 0.90, respectively). In comparison, the SVM model achieved 78% specificity and an AUC value of 0.85. The results are summarized in Table 4 and the ROC curves are shown in Fig. 5(b).

These findings confirmed that by accessing a large dataset, the deep learning model produced a higher number of representative segmentation features and exhibited greater overall accuracy for discriminating between malignant and benign breast lesions through mammography compared to standard models. Furthermore, the discriminative power of the deep learning model was greatest if a combinatorial approach was applied to characterize microcalcifications and breast masses simultaneously.

## Discussion

Mammography is considered the primary imaging modality for early detection and treatment of breast cancer; however, achieving accurate diagnoses through mammography is often challenging for radiologists due to the difficulty of distinguishing the features of malignant symptoms in images^42^,^43^,^44^. Consequently, considerable research is being undertaken to develop computer-based applications including various classification models to overcome these challenges^10^,^12^,^14^,^45^,^46^,^47^.

Microcalcifications are highly correlated with breast cancer^2^,^3^,^7^, therefore, the aim of this investigation was to evaluate the performance of an innovative deep learning model for classifying breast lesions. The results demonstrated that deep learning not only enabled accurate segmentation of microcalcifications but also provided an efficient analysis of their characteristics, leading to a marked improvement in discriminating between benign and malignant breast lesions compared to more standard SVM, KNN and LDA methods. This may have particular significance for cases in which microcalcifications are the only indicator of malignant lesions^4^,^11^,^48^,^49^.

Deep learning-based models employing large sample sets show greater discriminative performance in classifying microcalcifications through mammography compared to other machine learning methods. Compared to other methods, deep learning-based models provide a higher number of image segmentation features and help enhance the diagnostic accuracy through comprehensive characterization of these features. The discriminative power of deep learning can be increased by adopting a combinatorial approach to classify microcalcifications and masses simultaneously. Our results suggest that deep learning based-models on large datasets are promising in the earlier detection and treatment of breast cancer by identifying microcalcifications on mammograms.

Breast masses are also know to exhibit distinct features that vary from benign to malignant lesions^2^; however, machine-based methods are generally based on detecting microcalcifications or breast masses in isolation. In contrast, reports on methods that detect microcalcifications and masses simultaneously are scarce. In this study, we carried out a provisional and innovative trial using our deep learning-based model to distinguish both features in combination. The results showed that this combinatorial approach enhanced the diagnostic sensitivity of the model in patients presenting with both microcalcifications and masses. This implied that deep learning may offer an advanced statistical method for differentiating mammographic microcalcifications with greater accuracy and sensitivity, both in the presence or absence of breast masses. Not only could this facilitate earlier and more accurate classification of breast cancer, but also improve prognosis through timely treatment in malignant cases. It may also help avoid unnecessary surgical procedures, including total resection, and psychological and physiological pain in benign cases.

However, the current study suffered from the following limitations. First, the testing dataset should to be expended to provide more benign and malignant samples in order to achieve higher statistical power. In addition, by increasing the number of cases with breast masses, either alone or with microcalcifications, would allow deeper examination of the combinatorial approach and facilitate establishing the optimal diagnostic performance of our model and its potential value in future applications. Second, the features investigated in present study may not so sufficient enough to fully characterize microcalcifications, future studies will extract more. By selecting the most discriminative subset of them and optimizing the selection of various features, it helps improve the performance of deep learning in the classification stage. The current study was aiming to employ powerful deep learning based classifier to discriminate breast lesions by microcalcifications with or without the combined analysis of masses. With the settlement of problems addressed before, the nice performance of our trial in using deep learning opens a way to aid radiologist’s diagnostic performance. It further facilitates the systematical investigation of breast cancer for early detection, diagnosis and clinical management.

## Methods

### Participant population

We retrospectively reviewed mammograms from 1204 female patients histopathologically diagnosed with benign or malignant breast lesions at the SunYat-sen University Cancer Center (Guangzhou, China) and Nanhai Affiliated Hospital of Southern Medical University (Foshan, China) between May 2011 and March 2015. The sample comprised of 774 benign and 430 malignant breast lesions. All patients underwent molybdenum targeted mammography. Identified lesions were histopathologically confirmed as benign or malignant by performing open surgical biopsy or fine needle biopsy. The sample was divided into two groups: the training group comprised of images from 1000 randomly selected patients admitted between May 2011 and March 2015 (range, 26–75 years); the test group comprised of images from 204 randomly selected patients admitted between October 2013 and March 2015 (range, 28–75 years). All experimental protocols were approved by the Ethics Committee of the SunYat-sen University Cancer Center and the Ethics Committee of the Nanhai Affiliated Hospital of Southern Medical University, and were conducted in accordance with the Good Clinical Practice guideline. Informed consent was obtained from each patient for their consent to have their information used in research without affecting their treatment option or violating their privacy.

### Imaging and analysis

Images were obtained on a GE Senographe DS mammography system and a Siemens Mammomat Inspiration mammography system. Craniocaudal (CC) and mediolateral oblique (MLO) projections were obtained for each breast. All images were digitized at a resolution of 1024 × 1024 pixels and at 8-bit gray scale level. Taking the raw image directly may bring in a large bias due to image deformation, uniform background illumination, uneven imaging angle and position. Such problems may deteriorate the classification performance. To alleviate the problems, this study used various types of features that were widely used in researches on breast lesions as input data instead of original images^34^,^40^,^41^. We not only considered the features invariant to rotation, but also the features invariant to rotation, scaling, and translation. A previously reported computerized segmentation approach^29^ was used to extract any suspicious microcalcifications and masses from each image. Data about microcalcifications and suspicious breast masses were extracted through image segmentation. Both statistical and textural features were used to classify image features and obtain comprehensive characterization of microcalcifications and breast masses. A total of 41 quantitative measurements were recorded for each patient. Fifteen microcalcifications features and twenty-six breast masses features, estimated from the region of interests, were selected instead of original images as the input data for SAE model. The extracted features from mammograms aimed to provide comprehensive characterization of the image as much as possible. They consisted of intensity, statistic, shape and texture features. These features were extensively reported and tested widely in researches on breast lesions^18^,^19^,^23^,^24^,^34^,^40^,^41^. The 15 microcalcifications features were selected to describe different dimensional aspects of microcalcifications, including one-dimensional shape features (average diameter), two-dimensional morphological features (microcalcifications area), fractal dimensional features (microcalcifications density, circularity proportion, solidity, sandy microcalcification, spiculation, volume ratio), gray level intensity statistics features (mean gray value), and statistic feature (microcalcifications number, circularity, linear microcalcification). The 26 breast masses features also characterized different aspects of masses, including morphological features (breast masses area), fractal dimensional features (solidity, elongation, axis ratio, heterogeneity, spiculation, volume ratio, convexity), texture features (mean gray, maximum gray, gray relativity, entropy, inverse difference entropy, difference entropy, correlation, difference variance, sum average, sum variance, energy, mutual information). Detailed information about the features was provided in the Appendix File S1. Once the comprehensive characterization for each lesion done, its feature description was feed into the deep learning model to classify its type into benign or malignant.

### Deep learning model

Deep learning is a machine learning model with multiple hidden layers that learns inherent rules and features of large data sets. A stacked autoencoder (SAE) creates a deep network by stacking multiple autoencoders hierarchically^31^,^34^,^35^. Each autoencoder is a neural network (NN) that attempts to reproduce its input; the output of each autoencoder is used as the training set for the next autoencoder. More specifically, in an SAE with^*n*^ layers, the first layer is trained as an autoencoder to obtain the first hidden layer, and the output of the ^*k*^th hidden layer is used as the input of the ^(*k*+1)^th hidden layer.

In this study, 15 microcalcifications features and 26 breast masses features were selected instead of original images as the input data for SAE model, respectively. The SAE model was trained in a layer-wise greedy fashion to learn low-level features of microcalcifications from input data according to the following mathematical procedures:

Training samples were denoted as 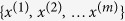; an autoencoder encoded input*x*^(*i*)^ to a hidden representation 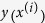 through a deterministic mapping function:


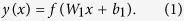


Conversely, the autoencoder decoded the representation 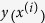 back into a reconstruction through a second deterministic mapping function:


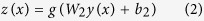


where *W*_1_ is a weight matrix, *W*_2_ is a decoding matrix, *b*_1_ is an encoding bias vector, and *b*_2_ is a decoding bias vector.

A logistic sigmoid function: 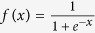 and 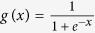 was used in this study.

The objective of an autoencoder was to minimize the reconstruction error by applying the following formula:


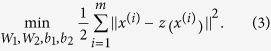


The encoding procedure was carried out from the first layer to the last layer by the following formulas:


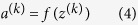



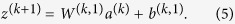


The decoding procedure was calculated from the last layer to the first layer by the following formulas:


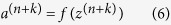



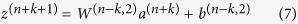


where 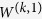 is a weight matrix of the *k*^th^ autoencoder, *W*^(*k*,2)^ is a decoding matrix of the *k*^th^ autoencoder, 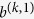
is an encoding bias vector of the *k*^th^ autoencoder, and 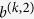 is a decoding bias vector of the *k*^th^ autoencoder, 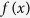 is sigmoid function, 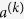 is sigmoid value.

We added a softmax classifier on the top layer of the SAE network to create the deep learning model for analyzing breast lesions^50^,^51^,^52^,^53^,^54^,^55^.

## Additional Information

**How to cite this article**: Wang, J. *et al.* Discrimination of Breast Cancer with Microcalcifications on Mammography by Deep Learning. *Sci. Rep.* **6**, 27327; doi: 10.1038/srep27327 (2016).

## Supplementary Material

Supplementary Information

## Figures and Tables

**Table 1:** Distributions of histopathological characteristics of breast lesions for both groups.

	Training group		Testing group	
	Number	Percentage	Number	Percentage
**Malignant lesions**	323	32.3	107	52.5
Invasive ductal carcinoma	222	22.2	86	42.2
Introductal carcinoma	8	0.8	2	0.9
Ductal carcinoma *in situ*	85	8.5	18	8.8
Mucinous carcinoma	4	0.4	0	0
Others	4	0.4	1	0.5
**Benign lesions**	677	67.7	97	47.6
Fibroadenoma	71	7.1	11	5.3
Fibrocystic changes	491	49.1	58	28.4
Intraductal papilloma	11	1.1	2	0.9
Hyperplasia	6	0.6	2	0.9
Phyllodes tumor	8	0.8	0	0
Inflammation	2	0.2	1	0.5
Follow-up	88	8.8	23	11.3
**Microcalcifications only**	623	62.3	110	53.9
**Masses only**	221	22.1	35	17.2
**Microcalcifications and masses**	156	15.6	59	28.9

Note—The follow-up period was at least two years. Only both systematic clinical examination and mammogram showed no malignant findings to the suspicious benign-appearing lesion in this period, can the patient be admitted into the benign group.

**Table 2:** Diagnostic performances of different classification models through microcalcification features (15 features).

	Test Dataset				Training Dataset
	accuracy	sensitivity	specificity	AUC	mean ± std (Accuracy)
SVM	85.8%	0.93	0.79	0.85	0.79 ± 0.07
KNN (N = 8)	83.8%	0.95	0.74	0.84	0.77 ± 0.07
LDA	58.8%	0.63	0.55	0.59	0.61 ± 0.05
SAE	**87.3%**	**0.93**	**0.82**	**0.87**	**0.82** ± **0.05**

The proposed SAE achieved superior performance in terms of the four measurements. The best measurements were highlighted in bold.

**Table 3:** Diagnostic performances of different classification models through mass features (26 features).

	Test Dataset				Training Dataset
	accuracy	sensitivity	specificity	AUC	mean ± std (Accuracy)
SVM	61.3%	1.00	0.26	0.60	0.68 ± 0.11
KNN (N = 8)	58.8%	1.00	0.21	0.57	0.71 ± 0.11
LDA	53.4%	0.99	0.12	0.52	0.67 ± 0.13
SAE	**61.3%**	**0.99**	**0.27**	**0.61**	**0.71** ± **0.12**

The proposed SAE achieved superior performance in terms of the four measurements. The best measurements were highlighted in bold.

**Table 4:** Diagnostic performances of different classification models through microcalcifications and mass features in combination (41 features).

	Test Dataset				Training Dataset
	accuracy	sensitivity	specificity	AUC	mean ± std (Accuracy)
SVM	85.8%	0.95	0.78	0.85	0.79 ± 0.07
KNN (N = 6)	84.3%	0.94	0.76	0.83	0.77 ± 0.06
LDA	74.0%	0.84	0.65	0.74	0.69 ± 0.07
SAE	**89.7%**	**0.89**	**0.90**	**0.90**	**0.85** ± **0.06**

The proposed SAE achieved superior performance in terms of the four measurements. The best measurements were highlighted in bold.
