# Supplementary material for: Discrimination of Breast Cancer with Microcalcifications on Mammography by Deep Learning
Source: Sci Rep. 2016 Jun 7;6:27327. doi: 10.1038/srep27327 (PMC4895132; doi:10.1038/srep27327)
Supplement: Supplementary Information [file srep27327-s1.doc]

# *Appendix*

**Discrimination of Breast Cancer with Microcalcifications on** **Mammography by Deep Learning**

**Jinhua Wang^1,*^, Xi Yang^2,*^, Hongmin Cai^2^, Wanchang Tan^1^, Cangzheng Jin^1^, Li Li^3^**

^1^Department of Radiology, Affiliated Nanhai Hospital of Southern Medical University, Foshan 528200, Guangdong, China.

^2^School of Computer Science and Engineering, South China University of Technology, Guangzhou 510006, Guangdong, China.

^3^Sun Yat-sen University Cancer Center; State Key Laboratory of Oncology in South China; Collaborative Innovation Center for Cancer Medicine; Guangzhou 510060, Guangdong, China

^*^ These authors contributed equally to this work.

Correspondence and requests for materials should be addressed to L.L. ([li2@mail.sysu.edu.cn](mailto:li2@mail.sysu.edu.cn)) or C.H. ([hmcai@scut.edu.cn](mailto:hmcai@scut.edu.cn))

## File S.1 Quantitative measurements of breast lesions

To achieve comprehensive morphological characterization of the microcalcifications, 38 parameters were automatically computed based on image segmentation. The detailed definitions of the parameters are listed as below:

***Gray-level co-occurrence matrix (GLCM) and features extracted from GLCM***

Image properties were estimated through a spatial gray-level co-occurrence matrix (GLCM) related to second-order statistics. Each element (*i*,*j*) in a GLCM specifies the number of times a pixel with gray-level value *i* occurs adjacent to a pixel with value *j* at a given offset$(\Delta x,\Delta y)$. The GLCM elements over an image S is given by the following formula:

$$p(i,j)=\frac{\#\{[(x,y),(x+\Delta x,y+\Delta y)]\in S|f(x,y)=i,f(x+\Delta x,y+\Delta y)=j\}}{\#S}$$

where represents the number of specific pixel-pairs.

If *N* is the number of distinct gray-levels of an image, each element is denoted as follows:

$p_{x}(i)=\sum_{j=1}^{N} p(i,j)$, $i=1,2,\cdots,N$

$p_{y}(j)=\sum_{i=1}^{N} p(i,j)$, $j=1,2,\cdots,N$

$p_{x+y}(k)=\sum_{i=1}^{N} \sum_{j=1}^{N} p(i,j)$, $k=i+j=2,3,\cdots,2N$

$p_{x-y}(k)=\sum_{i=1}^{N} \sum_{j=1}^{N} p(i,j)$, $k=\left|i-j\right|=0,1,\cdots N-1$

The thirteen texture features are then calculated as follows:

*f*_2_: **Contrast**: $f_{1}=\sum_{k=0}^{N-1} k^{2}p_{x-y}(k)$

*f*_5_: **Sum Average**: $f_{5}=\sum_{k=2}^{2N} kp_{x+y}(k)$

*f*_6_: **Sum Variance**: $f_{6}=\sum_{k=2}^{2N} (k-f_{5})^{2}p_{x+y}(k)$

*f*_7_: **Sum Entropy**: $f_{7}=-\sum_{k=2}^{2N} p_{x+y}(k)\log(p_{x+y}(k))$

*f*_8_: **Entropy**: $f_{8}=-\sum_{i} \sum_{j} p(i,j)\log(p(i,j))$

*f*_10_: **Difference Variance:** $f_{10}=\sum_{k=0}^{N-1} (k-d)p_{x-y}(k)$

where $d$is the mean of $p_{x-y}$.

*f*_11_: **Difference Entropy**: $f_{11}=-\sum_{k=0}^{N-1} p_{x-y}(k)\log(p_{x-y}(k))$

*p*_1_: **Compactness**: $p_{1}=\frac{P^{2}}{4\pi S}$

where $P$and$S$ are the perimeter length and area, respectively, for a given breast MRI lesion contour.

*p*_2_: **Spiculation**: $p_{2}=\frac{1}{N}\sum_{i=1}^{N} \left|r_{i}-r_{i+1}\right|$

where $N$is the number of pixels on the lesion contour and $r_{i}$ is the individual radial length.

*p*_3_: **Extent**: $p_{3}=\frac{S}{S_{\text{box}}}$

where $S_{box}$ is the area of the smallest rectangle containing the given lesion

contour.

*p*_4_: **Elongation**: $p_{4}=\frac{\min(H,L)}{\max(H,L)}$

where $H$ and $L$ are the vertical and horizontal lengths, respectively, of the smallest rectangle containing the given lesion contour.

*p*_5_: **Solidity**:

where $S_{convex}$ is the area of the smallest convex polygon that can contain the given lesion contour.

*p*_6_: **Circularity**: $p_{6}=\frac{1}{N-1}\sum_{i=1}^{N} r_{i}-\mu_{r}$

where $\mu_{r}$ is the average of$r_{i}$.

*p*_7_: **Entropy of radial length distribution**: $p_{7}=-\sum p(r_{i})\log(p(r_{i}))$

where $p(r_{i})$ is the probability density of a given$r_{i}$.

**Mutual information:** degree of similarity about *GLCM****.***

**Heterogeneity**: fraction of pixels that deviates more than a certain range (10%

default) from the average intensity.

**Fractal dimension**: Minkowski dimension of the boundary of the node, computed by box-counting method.

**Minkowski dimension**: a way of determining the [fractal dimension](http://en.wikipedia.org/wiki/Fractal_dimension) of set S in

an [Euclidean space](http://en.wikipedia.org/wiki/Euclidean_space) $R^{n}$. It is estimated by limit of $\lim_{\varepsilon\to0} \frac{N_{\varepsilon}}{1/\varepsilon}$, where $N(\varepsilon)$is the number of boxes of side length $\varepsilon$required to cover the set S.

**Area**: pixel numbers of the lesion region.

**Eccentricity**: scalar that specifies the eccentricity of the ellipse that has the

same second-moments as the region. The eccentricity is the ratio of the distance between the foci of the ellipse and its major axis length.

**Microcalcifications density**:

where $S_{convex}$ is the area of the smallest convex polygon that can contain the

given lesion contour, is the number of microcalcification of the smallest convex polygon that can contain the given lesion contour.

**Energy**: the feature reflects the degree of gray uniformity and texture

thickness. The big energy value represents a more uniform and regular pattern of texture.

**Circularity microcalcification:** the number of circularity microcalcification.

**Circularity proportion**: the proportion of the circularity microcalcifications in the number of microcalcifications.

**Sandy**: the proportion of the microcalcifications whose diameter <0.05mm.

**Speculation**: the feature of calcification boundary. The smaller the value, the

coarser boundary.
